# Serum bilirubin concentration is modified by *UGT1A1* Haplotypes and influences risk of Type-2 diabetes in the Norfolk Island genetic isolate

**DOI:** 10.1186/s12863-015-0291-z

**Published:** 2015-12-02

**Authors:** M. C. Benton, R. A. Lea, D. Macartney-Coxson, C. Bellis, M. A. Carless, J. E. Curran, M. Hanna, D. Eccles, G. K. Chambers, J. Blangero, L. R. Griffiths

**Affiliations:** Genomics Research Centre, Institute of Health and Biomedical Innovation, Queensland University of Technology, Kelvin Grove, QLD 4059 Australia; Kenepuru Science Centre, Institute of Environmental Science and Research, Wellington, 5240 New Zealand; Texas Biomedical Research Institute, San Antonio, TX 78227-5301 USA; School of Biological Sciences, Victoria University of Wellington, Wellington, 6140 New Zealand; South Texas Diabetes and Obesity Institute, University of Texas, Rio Grande Valley School of Medicine, Brownsville, TX 78520 USA

**Keywords:** Norfolk Island, GWAS, Bilirubin, type-2 diabetes, *UGT1A1*

## Abstract

**Background:**

Located in the Pacific Ocean between Australia and New Zealand, the unique population isolate of Norfolk Island has been shown to exhibit increased prevalence of metabolic disorders (type-2 diabetes, cardiovascular disease) compared to mainland Australia. We investigated this well-established genetic isolate, utilising its unique genomic structure to increase the ability to detect related genetic markers. A pedigree-based genome-wide association study of 16 routinely collected blood-based clinical traits in 382 Norfolk Island individuals was performed.

**Results:**

A striking association peak was located at chromosome 2q37.1 for both total bilirubin and direct bilirubin, with 29 SNPs reaching statistical significance (*P* < 1.84 × 10^−7^). Strong linkage disequilibrium was observed across a 200 kb region spanning the UDP-glucuronosyltransferase family, including *UGT1A1*, an enzyme known to metabolise bilirubin. Given the epidemiological literature suggesting negative association between CVD-risk and serum bilirubin we further explored potential associations using stepwise multivariate regression, revealing significant association between direct bilirubin concentration and type-2 diabetes risk. In the Norfolk Island cohort increased direct bilirubin was associated with a 28 % reduction in type-2 diabetes risk (OR: 0.72, 95 % CI: 0.57-0.91, *P* = 0.005). When adjusted for genotypic effects the overall model was validated, with the adjusted model predicting a 30 % reduction in type-2 diabetes risk with increasing direct bilirubin concentrations (OR: 0.70, 95 % CI: 0.53-0.89, *P* = 0.0001).

**Conclusions:**

In summary, a pedigree-based GWAS of blood-based clinical traits in the Norfolk Island population has identified variants within the UDPGT family directly associated with serum bilirubin levels, which is in turn implicated with reduced risk of developing type-2 diabetes within this population.

**Electronic supplementary material:**

The online version of this article (doi:10.1186/s12863-015-0291-z) contains supplementary material, which is available to authorized users.

## Background

This study examined a large multi-generational pedigree from the isolated population of Norfolk Island to identify genomic variants (SNPs – single nucleotide polymorphisms) associated with routinely collected blood-based clinical traits. The Norfolk Island population is a genetic isolate with strong family groups and a well-documented family genealogy [1]. Norfolk Island is a small volcanic island located in the Pacific Ocean between Australia (about 1600 km north-east of Sydney) and New Zealand (1077 km north-west of Auckland). Alongside geographic isolation, a unique history has shaped the genomic architecture of the current pedigree members resulting in an admixed population with both European and Polynesian ancestry [2]. Recent estimation of the admixture in the Norfolk Island cohort reported 88 % European ancestry and 12 % Polynesian ancestry [2].

To date the Norfolk Island Health Study (NIHS) has collected data and samples for 1199 Norfolk Islanders, 52 % (*N* = 624) of whom were found to have direct links to the original founders. Using this in-depth genealogical information a large multi-generational Norfolk pedigree was reconstructed [1]. Several studies have established admixture scores and presence of founder effects within the Norfolk Island pedigree [1–3] and the pedigree has been shown to have sufficient power to detect genetic loci influencing complex traits via linkage and association [4–7].

The Norfolk Island population has high rates of metabolic syndrome [7] and cardiovascular related risk factor traits, especially obesity, compared to mainland Australia. Research on the Norfolk pedigree has shown that traits for obesity, dyslipidaemia, blood glucose and hypertension exhibit a substantial genetic component, with heritability estimates ranging from 30 % for systolic blood pressure (SBP) to 63 % for low density lipoproteins (LDL) cholesterol [1, 4, 5]. In addition, factor analysis identified “composite” phenotypes with high heritability [5], suggesting that common gene(s) underlie cardiovascular disease-related phenotypes. Furthermore, genetic linkage analysis in the Norfolk Island pedigree has successfully identified previously documented regions associated with cardiovascular disease risk traits, the most significant being for SBP on chromosome 1 (1p36) [4].

Reported rates of type-2 diabetes within the Norfolk Island population are similar to mainland Australia (4-8 %). However, a significantly higher proportion of individuals had fasting blood glucose in excess of normal ranges (>5 mmol/L), suggesting a high prevalence of pre-diabetes and possible under-diagnosis of type-2 diabetes [4, 8]. Additionally, clinical diagnosis of type-2 diabetes using AUSDRISK [9] identified that 42 % of the Norfolk Island population were at high-risk of developing the disease [7].

Bilirubin is a component of haemoglobin, formed during metabolic breakdown in the liver. Total serum bilirubin measures both water-soluble (direct-) and fat-soluble (indirect-) bilirubin. Bilirubin is also a potent antioxidant and as such has a vital role in the protection of the body against reactive oxygen species [10–12]. Numerous epidemiological analyses have reported strong negative associations between CVD-risk and serum bilirubin levels. Very few studies investigating the link between type-2 diabetes and serum bilirubin concentration have been conducted [13], although recently an association with mortality in a type-2 diabetic cohort was observed [14]. Serum bilirubin concentration has been shown to be tightly regulated by the UDP-glucuronosyltransferase (UDPGT) enzyme family, with several large GWAS and linkage studies identifying variants within UGT1A in particular [15–18]. This is suggestive of a potentially heritable metabolic disease factor, for which a recent study provides further supportive evidence; a Mendelian randomization study exploring total bilirubin levels in a prospective study found further evidence for a protective role in type-2 diabetes [19].

The aim of this study was to update the previously calculated heritabilities for a range of blood-based traits relating to CVD risk in the Norfolk Island cohort and to perform genome-wide association studies (GWASs) of the heritable traits using a pedigree-based approach.

## Results

### Heritability of individual metabolic traits

A description of the blood-based clinical traits investigated in this study, including summary statistics, is shown in Additional file 1. The latest pedigree relationship information and GenABEL were used to calculate heritability (h^2^) statistics for all traits profiled in the Norfolk Island cohort. In total, 16 traits (out of 19) yielded statistically significant h^2^ values ranging from 0.225 – 0.563 (nominal *P* < 0.05). The average heritability was 0.39 and 8 traits exhibited a higher than average heritability (total protein, globin, total bilirubin, LDL-C, cholesterol, alkaline phosphatase, and urea) the most heritable trait being total protein (h^2^ = 0.563, *P* = 2.26 × 10^−4^). A summary of all significantly heritable major blood-based clinical traits is shown in Table 1.

**Table 1:** Significantly heritable metabolic traits in the Norfolk Island population

Trait	h^2^	*P* value
Total protein	0.563	2.26E-04
Globin	0.525	3.36E-04
Total bilirubin	0.502	4.45E-05
LDL-C	0.454	7.51E-05
Cholesterol	0.426	8.41E-05
Chlorine	0.426	8.41E-05
Alkaline phosphatase	0.425	6.10E-04
Urea	0.424	7.87E-04
GGT	0.369	3.02E-03
Albumin	0.358	9.01E-03
Uric Acid	0.350	2.17E-03
HDL-C	0.348	1.58E-02
Direct Bilirubin	0.327	3.30E-02
Creatinine	0.257	2.43E-02
AST	0.251	3.81E-02
Cholesterol/HDL-C ratio	0.225	2.42E-02

### GWAS of metabolic traits

All 16 heritable blood-based clinical traits were screened for association separately; individual trait GWAS Manhattan plots can be viewed in Additional file 2. There were 2 traits with robustly associated clusters (i.e. SNPs in close proximity to each other); total bilirubin and direct bilirubin. It should be noted that a number of SNPs passed the adjusted significance threshold for liver function traits (i.e. GGT, AST, ADH). These traits exhibited numerous SNPs passing *M*_*eff*_ adjustment, however robust 'peaks'/clusters of SNPs were not observed.

### Exploration of the bilirubin association on chromosome 2q37.1

**Fig. 1:**
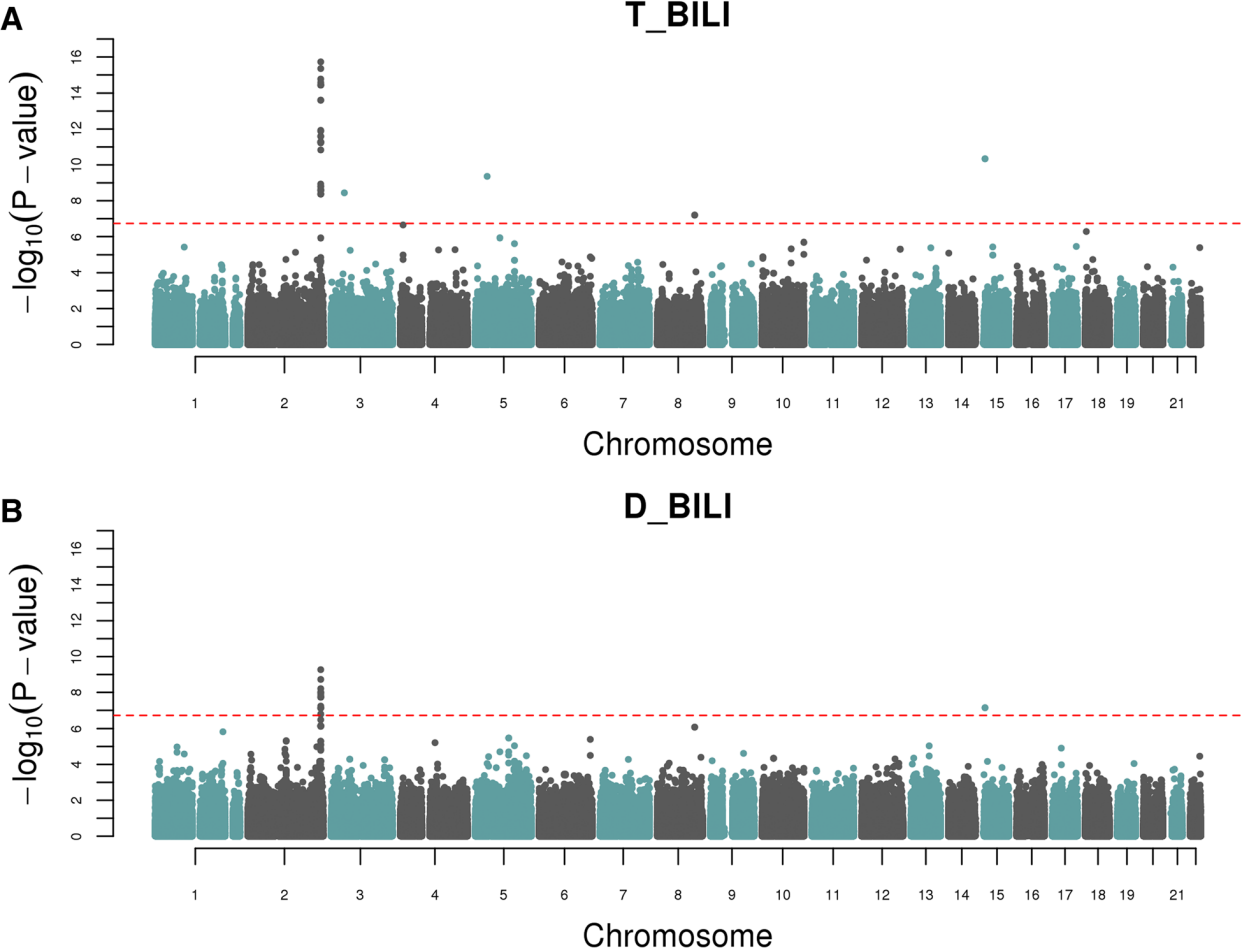
GWAS Manhattan plots for; **a** Total Serum Bilirubin, and **b** Direct Serum Bilirubin. *M*_*eff*_ adjusted correction threshold of 1.84 × 10^−7^ is indicated by the horizontal dashed line

**Fig. 2:**
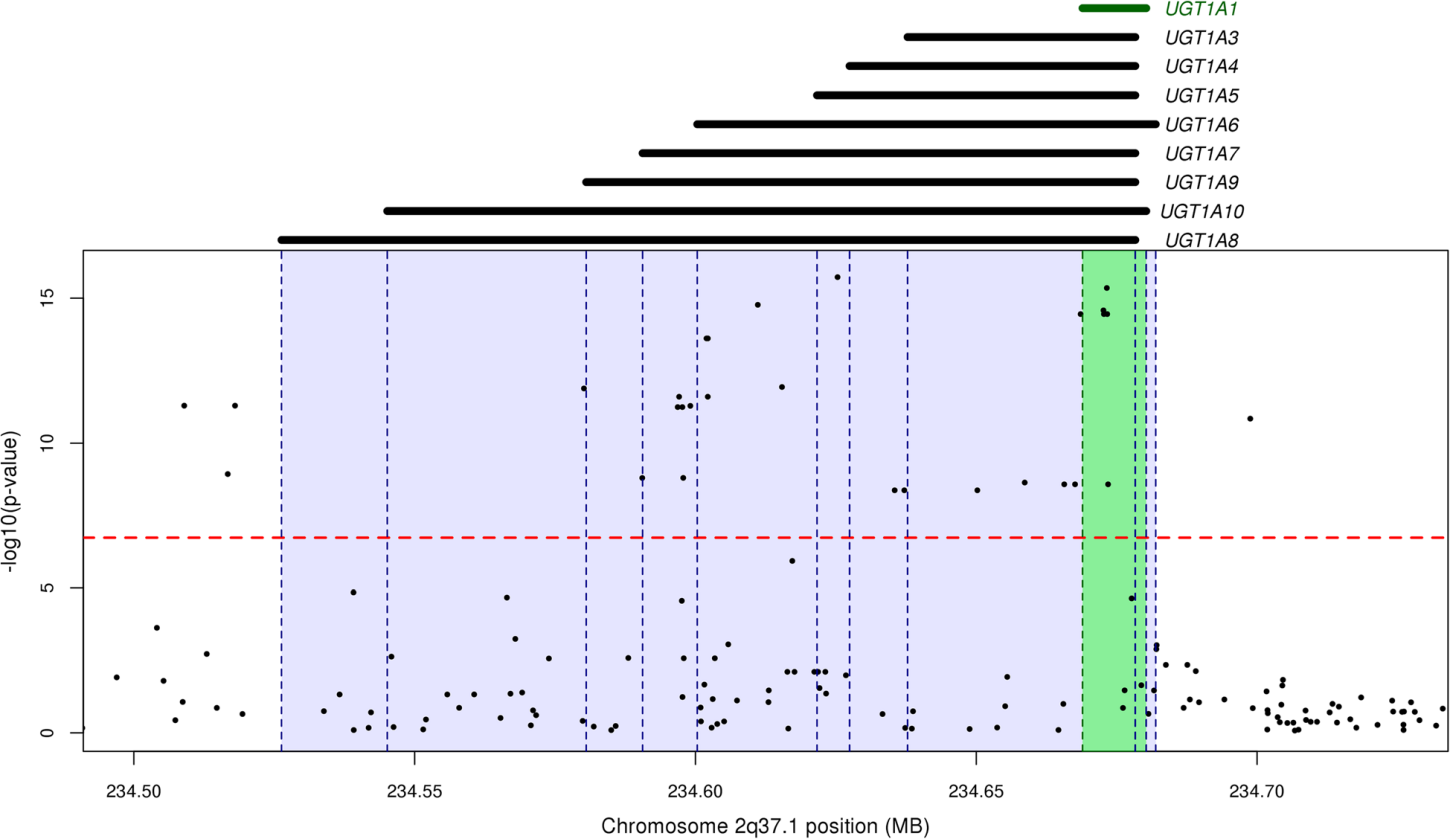
Magnified view displaying genomic structure of the UDP-glucuronosyltransferase gene family located on 2q37.1. All SNPs within this region that were tiled on the Illumina Human 610quad BeadChip are displayed. *M*_*eff*_ adjusted correction threshold of 1.84 × 10^−7^ is indicated by the horizontal dashed line. Known gene isoforms are indicated by dashed vertical lines and labelled at the top of the plot. The bilirubin metabolising gene, UGT1A1, is shown highlighted in green

The strongest observed association was seen between a cluster of 29 SNPs on chromosome 2q37.1 passing a *M*_*eff*_ adjusted threshold and total serum bilirubin (Fig. 1a, Table 2). The most robustly associated SNP was rs6744284 (P = 1.87 × 10^−16^). A weaker association was observed for the same cluster of SNPs on chromosome 2q37.1 with direct serum bilirubin levels (Fig. 1b). These 29 SNPs span a region of 189.8 kb, and lie directly on top of a complex locus that codes numerous isoforms of the UDP-glucuronosyltransferase (UGT) family (Fig. 2).

**Table 2:** Top *M*_*eff*_ corrected SNPs associated with total serum bilirubin

SNP	CHR	Position	A1	A2	effB	se_effB	chi2.1df	*P* value
rs6744284	2	234625297	C	T	3.278	0.395	68.90	1.87E-16
rs3771341	2	234673239	C	T	3.235	0.395	67.17	4.43E-16
rs17863787	2	234611094	T	G	3.044	0.379	64.48	1.70E-15
rs6742078	2	234672639	G	T	3.025	0.379	63.60	2.64E-15
rs887829	2	234668570	G	A	3.011	0.379	63.01	3.55E-15
rs4148324	2	234672722	T	G	3.011	0.379	63.01	3.55E-15
rs4148325	2	234673309	C	T	3.011	0.379	63.01	3.55E-15
rs1105880	2	234601965	T	C	2.829	0.368	59.13	2.46E-14
rs1105879	2	234602202	T	G	2.829	0.368	59.13	2.46E-14
rs6725478	2	234615400	C	T	2.711	0.378	51.40	1.18E-12
rs2741045	2	234580140	C	T	2.907	0.406	51.17	1.32E-12
rs10168416	2	234597087	C	G	2.827	0.400	49.85	2.55E-12
rs2070959	2	234602191	A	G	2.827	0.400	49.85	2.55E-12
rs2741012	2	234508963	C	T	2.887	0.415	48.43	5.20E-12
rs2741027	2	234518011	G	A	2.887	0.415	48.43	5.20E-12
rs7608175	2	234599089	C	G	2.536	0.364	48.41	5.27E-12
rs10168155	2	234596836	C	T	2.530	0.364	48.21	5.82E-12
rs10171367	2	234597667	C	G	2.530	0.364	48.21	5.82E-12
rs2361502	2	234698790	T	C	2.590	0.380	46.37	1.46E-11
rs2925260	15	25869972	T	C	18.764	2.825	44.11	4.56E-11
rs2930593	15	25877815	C	A	18.764	2.825	44.11	4.56E-11
rs12654591	5	38271582	C	A	13.063	2.075	39.63	4.34E-10
rs2741023	2	234516714	G	A	2.446	0.399	37.67	1.17E-09
rs10179094	2	234597825	T	A	2.347	0.386	37.06	1.58E-09
rs7586110	2	234590527	T	G	2.344	0.385	37.05	1.59E-09
rs2221198	2	234658623	C	T	2.251	0.374	36.33	2.29E-09
rs4124874	2	234665659	A	C	2.194	0.365	36.05	2.63E-09
rs3755319	2	234667582	T	G	2.194	0.365	36.05	2.63E-09
rs4148326	2	234673462	T	C	2.194	0.365	36.05	2.63E-09
rs1876506	3	42547127	A	G	13.734	2.308	35.43	3.61E-09
rs4294999	2	234635467	A	G	2.155	0.364	35.11	4.23E-09
rs2008595	2	234637192	G	A	2.155	0.364	35.11	4.23E-09
rs4663963	2	234650193	T	G	2.155	0.364	35.11	4.23E-09
rs16892701	8	120525983	G	A	11.336	2.077	29.79	6.25E-08
rs16892482	8	120386382	A	G	11.326	2.077	29.74	6.41E-08

### LD block identification

**Fig. 3:**
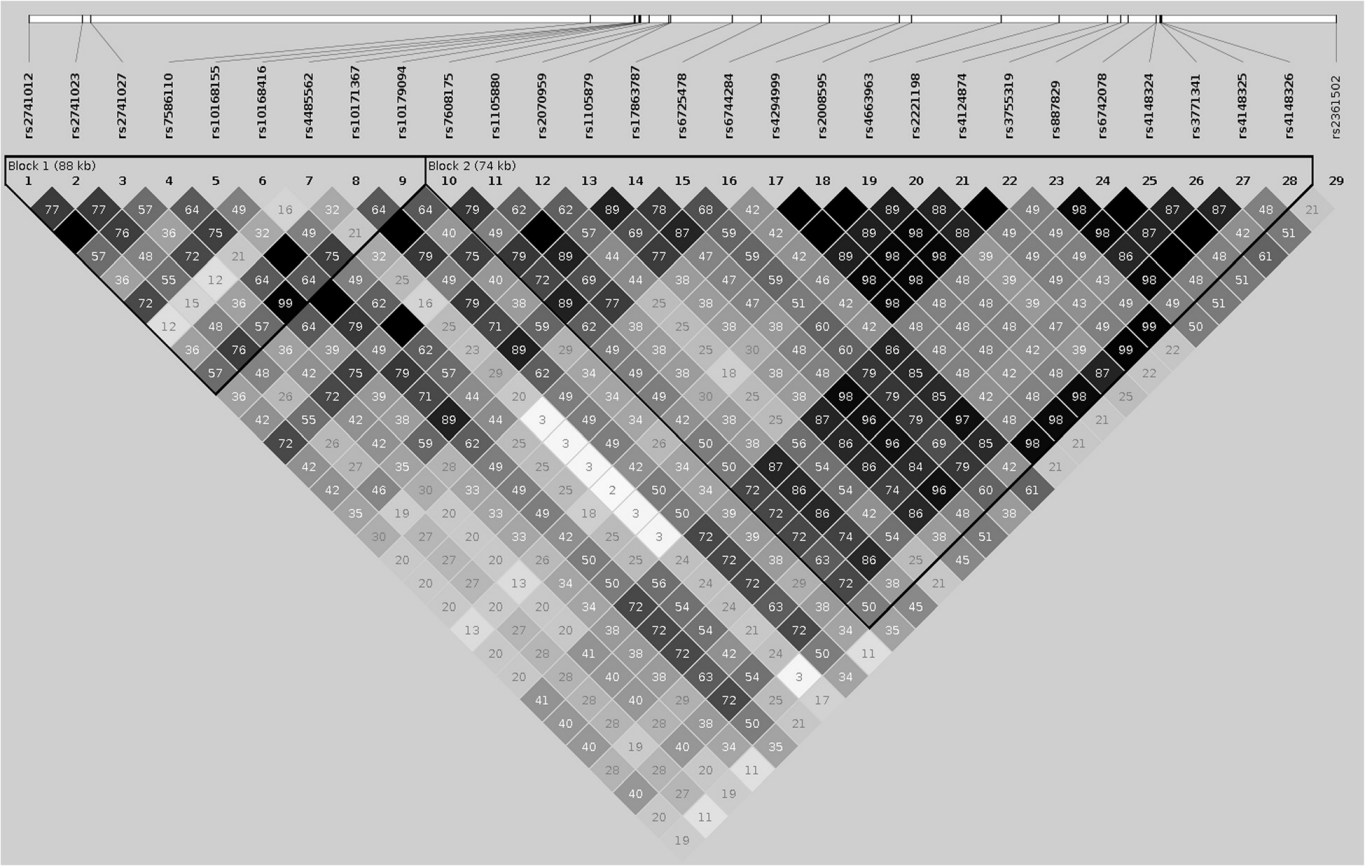
Linkage Disequilibrium plots for 29 SNPs contained within UDP-glucuronosyltransferase gene family. The 2 LD blocks are outlined in black; Block 1 spans SNPs 1–9, Block 2 spans SNPs 10–28. All SNP rs numbers are listed, with their chromosomal positioning relative to each other indicated at the top of the figure

Evidence of strong linkage disequilibrium (LD) across the 29 SNPs was observed in the Norfolk Island population (Fig. 3); summarised LD statistics for the 29 SNPs: r^2^ (min = 0.026, 1st Quartile = 0.33, median = 0.49, mean = 0.51, 3rd Quartile = 0.72, max = 1.00), D' (min = 0.24, 1st Quartile = 0.82, median = 0.90, mean = 0.89, 3rd Quartile = 1.00, max = 1.00).. Haploview analysis identified 2 LD blocks across the region; the first block contained 9 SNPs and spanned 88 kb, the second block consisted of 19 SNPs and spanned a region of 74 kb. Further analysis of LD across 3 separate HapMap populations was conducted to compare with that obtained in the Norfolk Island cohort; CEU (European), CHD (Chinese) and JPT (Japanese). Due to the use of different SNP arrays, 25 of the 29 SNPs were available across the 4 populations, thus the LD mapping was restricted to these 25 SNPs. The LD pattern for the Norfolk Island cohort was most similar to the CEU population, and extensively different from both of the Asian HapMap groups used (Additional file 3). LD appeared slightly stronger in the Norfolk Island SNPs than for CEU. Allele frequencies for the 25 SNPs in these 4 populations are detailed in Additional file 4.

### Haplotype mapping and association with bilirubin levels

Haploview association analysis was performed on the individual 29 SNP 'markers', minor allele frequencies (MAF) and association statistics are documented in Table 3 (for additional information see Additional file 5). All 29 SNPs exhibited significantly (*P* < 1.0 × 10^−4^) increased MAF in the high serum bilirubin group. The most significantly associated marker was rs17863787; the frequency of the ‘G’ allele was observed to be 62.3 % in those with high serum bilirubin and 24.9 % in those with normal serum bilirubin (*P* = 5.51 × 10^−17^).

**Table 3:** Haploview marker associations showing frequencies of the recessive alleles

SNP	Associated allele	High bili allele freq	Normal bili allele freq	OR (95 % CI)	Chi square	*P* value
rs2741012	T	0.415	0.188	0.33 (0.22 – 0.49)	31.93	1.60E-08
rs2741023	A	0.431	0.239	0.41 (0.28 – 0.61)	20.06	7.52E-06
rs2741027	A	0.415	0.188	0.33 (0.22 – 0.49)	31.93	1.60E-08
rs7586110	G	0.500	0.251	0.33 (0.23 – 0.49)	32.33	1.30E-08
rs10168155	T	0.654	0.326	0.26 (0.17 – 0.38)	48.96	2.62E-12
rs10168416	G	0.492	0.194	0.25 (0.17 – 0.37)	51.93	5.75E-13
rs4485562	G	0.800	0.626	0.42 (0.26 – 0.66)	14.44	1.00E-04
rs10171367	G	0.654	0.326	0.26 (0.17 – 0.38)	48.96	2.62E-12
rs10179094	A	0.500	0.249	0.33 (0.22 – 0.49)	32.83	1.01E-08
rs7608175	G	0.654	0.326	0.26 (0.17 – 0.38)	49.11	2.42E-12
rs1105880	C	0.646	0.271	0.20 (0.14 – 0.30)	68.04	1.60E-16
rs2070959	G	0.492	0.194	0.25 (0.17 – 0.37)	51.93	5.75E-13
rs1105879	G	0.646	0.271	0.20 (0.14 – 0.30)	68.04	1.60E-16
rs17863787	G	0.623	0.249	0.20 (0.13 – 0.30)	70.15	5.51E-17
rs6725478	T	0.631	0.300	0.25 (0.17 – 0.37)	51.58	6.88E-13
rs6744284	T	0.577	0.222	0.21 (0.14 – 0.31)	66.86	2.91E-16
rs4294999	G	0.715	0.429	0.30 (0.21 – 0.45)	35.46	2.61E-09
rs2008595	A	0.715	0.429	0.30 (0.21 – 0.45)	35.46	2.61E-09
rs4663963	G	0.715	0.429	0.30 (0.21 – 0.45)	35.46	2.61E-09
rs2221198	T	0.672	0.402	0.33 (0.22 – 0.49)	31.32	2.18E-08
rs4124874	C	0.715	0.427	0.30 (0.21 – 0.45)	35.86	2.12E-09
rs3755319	G	0.715	0.427	0.30 (0.21 – 0.45)	35.86	2.12E-09
rs887829	A	0.623	0.254	0.20 (0.14 – 0.30)	67.92	1.70E-16
rs6742078	T	0.623	0.253	0.20 (0.14 – 0.30)	68.23	1.46E-16
rs4148324	G	0.623	0.254	0.20 (0.14 – 0.30)	67.92	1.70E-16
rs3771341	T	0.577	0.227	0.22 (0.15 – 0.32)	64.55	9.40E-16
rs4148325	T	0.623	0.254	0.20 (0.14 – 0.30)	67.92	1.70E-16
rs4148326	C	0.715	0.427	0.30 (0.21 – 0.45)	35.86	2.12E-09
rs2361502	C	0.546	0.268	0.30 (0.21 – 0.45)	38.61	5.16E-10

Note: odds ratios are not adjusted for age and sex

**Fig. 4:**
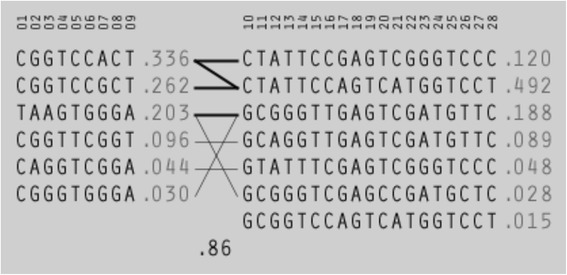
Haplotype structure across the two identified LD blocks in the Norfolk Island cohort UDP-glucuronosyltransferase gene family. Displayed haplotypes reside at >1 % frequency in the genotyped samples. Connecting lines represent haplotype combinations: thick lines represent haplotype combinations that reside at >10 %, thin lines >1 % of samples

To further investigate the association of genomic structure across the chr2q37.1 region with serum bilirubin, a haplotype association analysis was conducted in Haploview. There were a total of 6 haplotypes inferred for LD block 1 and 7 haplotypes for LD block 2 (Additional file 6); haplotypes present in >1 % of the total population are shown. The block 1 haplotype most significantly associated with the high bilirubin group was 'TAAGTGGGA', which is estimated to exist at 20.3 % in the total population. This haplotype was observed in 40.3 % of the high serum bilirubin group, and 17.2 % of the normal group (*P* = 4.59 × 10^−9^). The most abundant block 1 haplotype ('CGGTCCACT', 33.6 % of total population) was observed to be significantly associated with the normal serum bilirubin group; 36.9 % normal vs 19 % high (*P* = 9.31 × 10^−5^). The LD block 2 haplotype most significantly associated with high serum bilirubin was 'GGGCGTTGTGAGCTTGTTC'; which is estimated to be present in 18.8 % of the total population. This haplotype was observed in 43.5 % of the high serum bilirubin group, and 14.3 % of the normal group (*P* = 1.73 × 10^−14^). The most abundant block 2 haplotype ('CAAATCCACTGTACGTCCT', 49.2 % of total population) was observed to be significantly associated with the normal serum bilirubin group; 54.6 % normal vs 26.1 % high (*P* = 3.51x10^−9^). Frequency and combination of the block specific haplotypes is illustrated in Fig. 4.

Nine tagging SNPs were identified that capture the allelic variance of the 29 SNPs (Table 4); the tagging analysis captured all 29 alleles at r^2^ > = 0.8 which contains 100 % of alleles with mean r^2^ of 0.963. These SNPs could be used in future replication analyses to tag variation across the region in other populations.

**Table 4:** Haploview 'Tagger' analysis of the 29 GWAS associated chr2q37.1 SNPs identified 9 SNPs as tagging the allelic variation across the region

SNP tested	Alleles captured
rs4148325	rs3771341,rs887829,rs1105879,rs4148325,rs6742078,rs17863787,rs1105880,rs6744284,rs4148324
rs2008595	rs4294999,rs4148326,rs4663963,rs2221198,rs3755319,rs4124874,rs2008595
rs6725478	rs6725478,rs10168155,rs10171367,rs7608175
rs7586110	rs10179094,rs7586110
rs10168416	rs10168416,rs2070959
rs2741027	rs2741027,rs2741012
rs2741023	rs2741023
rs2361502	rs2361502
rs4485562	rs4485562

### Bilirubin correlations with clinical metabolic syndrome and cardiovascular disease

It is well established that serum bilirubin levels are inversely correlated with risk of developing cardiovascular disease [20–22]. Therefore this was investigated using the cardiovascular disease risk score previously calculated for the Norfolk Island population [7], along with potential relationships between other metabolic risk scores, including metabolic syndrome and type-2 diabetes (scores previously estimated [7]).

A significant inverse relationship was observed between total serum bilirubin and the clinical risk score for metabolic syndrome. Of the 592 individuals with available data 66 % had normal bilirubin levels and no metabolic syndrome, 11.5 % had high bilirubin levels and no metabolic syndrome, 25.3 % had normal bilirubin and metabolic syndrome, 1.2 % had high bilirubin and metabolic syndrome. A chi-squared contingency test followed by Fisher's exact showed that this was a significant observation; *χ*2 = 4.18 (*P* = 0.04), Fisher's Exact OR = 2.45 (*P* = 0.03). This correlation suggests that Norfolk Island individuals with higher serum bilirubin levels are less likely to develop metabolic syndrome.

Numerous studies have also attributed smoking behaviour to be associated with serum bilirubin levels [23–25]. This was tested in the Norfolk Island population using the students independent *t*-test, and revealed a significant difference in mean serum bilirubin levels between smokers (6.46 μmol/L) and non-smokers (8.12 μmol/L); t = 3.99 with P = 4.06 × 10^−5^.

To further examine potential relationships a series of t-tests between a variety of quantitative metabolic syndrome/cardiovascular disease traits and categorised serum bilirubin group were performed. There were a total of 9 significant (*P* < 0.05) trait correlations with categorised bilirubin level, these were; body mass index (BMI), body fat, cholesterol/HDL-C ratio, total cholesterol, hip circumference, LDL-C, type-2 diabetes risk score, total protein and triglycerides (Table 5). These findings highlight traits that are consistent with previous literature [26, 27].

**Table 5:** Metabolic trait correlation with serum bilirubin group

Trait	t	*P*	High bili mean	Normal bili mean
albumin	0.90	3.70E-01	41.75	41.43
Body Mass Index*	−1.91	3.00E-02	25.06	26.27
body fat*	−3.43	5.30E-04	26.63	30.67
cholesterol/HDL-C ratio*	−1.82	4.00E-02	3.94	4.29
cholesterol*	−2.71	4.00E-03	5.30	5.67
creatinine	0.15	8.80E-01	80.19	79.90
cardiovascular disease risk	0.40	6.90E-01	7.20	6.69
diastolic blood pressure	0.51	6.90E-01	77.69	76.68
globin	1.91	6.10E-02	30.29	29.34
HDL-C	0.62	5.30E-01	1.44	1.41
hip circumference*	−2.04	2.00E-02	99.42	101.92
Inbreeding	−0.42	3.40E-01	0.01	0.01
LDL-C*	−1.66	5.00E-02	2.66	2.86
mean arterial pressure	0.26	8.00E-01	95.01	94.44
Polynesian Admixture	−1.41	8.50E-02	0.21	0.25
pulse pressure	0.02	9.80E-01	52.31	52.26
systolic blood pressure	0.29	6.10E-01	130.00	128.95
type-2 diabetes risk*	−2.31	1.00E-02	9.19	11.22
total protein*	2.28	3.00E-02	72.10	70.79
triglycerides*	−2.15	2.00E-02	1.58	2.00
urea	−0.44	6.60E-01	5.49	5.58
uric acid	0.53	6.00E-01	0.35	0.34
waist circumference	−1.91	1.30E-01	85.04	87.62
waist-hip ratio	0.34	6.30E-01	0.86	0.86
weight	0.20	5.80E-01	75.79	75.31

* significant at *P* < 0.05

Body fat was observed to have the strongest correlation with serum bilirubin, with significantly reduced body fat composition in individuals who had high serum bilirubin levels. Unlike previous observations [20, 27, 28], cardiovascular disease risk score was not significantly reduced in those individuals with higher serum bilirubin, whereas, type-2 diabetes risk did show a significant reduction in the higher bilirubin group, consistent with previous literature [26, 29].

### Genotype effects on metabolic syndrome, type-2 diabetes and cardiovascular disease traits

To further explore the above approach, associations between the 29 significantly associated SNPs and metabolic traits other than serum bilirubin were explored. Traits which showed a significant (P < 0.05) correlation with total serum bilirubin (Table 5) were selected. Only one trait was observed which showed a significant association with any of the 29 markers, this was type-2 diabetes-risk when categorised: “low”; “intermediate”, and “high” [9]. Using a chi-squared test rs2741012 and rs2741027 were significantly associated with type-2 diabetes-risk (χ2 = 9.63, *P* = 0.0069). Again this was followed with a Fisher's Exact test which confirmed significance (*P* = 0.0081). The same observation with the minor allele and suggestive protection was observed.

To further investigate the above associations logistic regression was used to identify a model that predicts outcome (type-2 diabetes) from trait (bilirubin) and factors in potential modifiers (genotype). Logistic regression modelling identified direct bilirubin as being significantly associated with categorised type-2 diabetes risk (r^2^: 0.05, *p*-value: 0.005), suggesting that in the Norfolk Island cohort increased direct bilirubin was associated with a 28 % reduction in type-2 diabetes risk (OR:0.72, 95 % CI: 0.57-0.91). Based on a bi-directional stepwise regression model approach 2 of the 9 tagging SNPs remained significant; rs2741027 and rs6725478. These SNPs effectively tag the two major 'protective'/high bilirubin haplotypes. When included, the adjusted model remained significant (r^2^: 0.13, p-value: 0.0001) and confirmed the initial association; direct bilirubin (OR:0.70, 95 % CI: 0.53-0.89, p-value: 0.005): rs2741027 (OR:0.25, 95 % CI: 0.10-0.58, p-value: 0.002), rs6725478 (OR:0.27, 95 % CI: 0.10-0.63, p-value: 0.004). This indicates that when controlling for bilirubin levels genotype affects risk of type-2 diabetes within the Norfolk Island population. Therefore, inclusion of SNP genotypes when assessing the relationship between direct bilirubin and type-2 diabetes risk increases the accuracy of the 'risk' estimate within the Norfolk Island cohort.

### Functional Annotation of UDP-glucuronosyltransferase SNPs

Investigation of the 29 SNPs revealed several of potential functional interest (SNP annotation Table 6). Three SNPs are within the coding region of UGT1A6 (Table 6); rs1105880 (synonymous), rs1105879 and rs2070959 (non-synonymous). Further investigation with SNPnexus (http://www.snp-nexus.org/) revealed rs1105879 had a PolyPhen score of 'possibly damaging', indicating the usually conserved nature of the coded amino acid. Six SNPs were observed to reside within 5' prime untranslated regions (5'UTR); UGT1A1 (rs887829, rs3755319), UGT1A3 (rs2008589), UGT1A6 (rs7608175), UGT1A7 (rs7586110), and UGT1A9 (rs2741045).

**Table 6:** Functional annotation of the chr2q37.1 SNPs significantly associated with total serum bilirubin levels

RefSNP ID	NCBI gene ID	Gene symbol	Function class	Residue	Amino acid position
rs1105879	54578	UGT1A6	coding-nonsynonymous	S	183
rs2070959	54578	UGT1A6	coding-nonsynonymous	A	180
rs1105880	54578	UGT1A6	coding-synonymous	L	104
rs2008595	54659	UGT1A3	5'UTR	-	-
rs2741045	54600	UGT1A9	5'UTR	-	-
rs3755319	54658	UGT1A1	5'UTR	-	-
rs7586110	54577	UGT1A7	5'UTR	-	-
rs7608175	54578	UGT1A6	5'UTR	-	-
rs887829	54658	UGT1A1	5'UTR	-	-

SNPs displayed reside in regions other than introns

## Discussion

We have identified a significant genomic association at 2q37.1 in the region of the UDP-glucuronosyltransferase (UDPGT) enzyme family members, with direct and total serum bilirubin levels. Correlation analyses between metabolic syndrome related traits and serum bilirubin levels identified significant inverse relationships for numerous traits. Haplotype association testing revealed the presence of potentially protective haplotypes within the Norfolk Island population. Thus this study has identified a complex region which shows interplay between genomic and environmental conditions and has a large effect on overall serum bilirubin levels.

Previous literature has suggested a linkage between bilirubin and metabolic risk with clinical associations observed between cardiovascular disease risk, obesity and bilirubin concentrations [20–22, 27] and more recently metabolic syndrome [30–34]. Therefore, we investigated potential relationships between bilirubin and metabolic traits in the Norfolk Island cohort. An inverse correlation between serum bilirubin and several important metabolic traits was observed, with the most notable being metabolic syndrome and type-2 diabetes risk. Given that metabolic syndrome and type-2 diabetes increase cardiovascular disease risk it is consistent with the current body of literature which documents inverse association between high serum bilirubin and cardiovascular disease risk (review [26]).

Our analysis refined an association with serum bilirubin concentration to a 189.8 kb region on chromosome 2q37.1 with genotypic analyses revealing that the level of serum bilirubin was greatly increased in individuals with the rare allele. This region encodes one of the major drug metabolising families (UDP-glucuronosyltransferase, UDPGT) [35–37]; there are 9 documented UDPGT isoforms; UGT1A1, UGT1A3, UGT1A4, UGT1A5, UGT1A6, UGT1A7, UGT1A8, UGT1A9 and UGT1A10 (Fig. 2). UGT1A1 is well known to preferentially metabolise bilirubin and has been previously mapped in linkage and GWAS studies [16–18, 38–43]. UGT1A3 and UGT1A4 also have been shown to have potential action with bilirubin [37]. However all gene family members, including UGT1A1, exhibit affinity for numerous substrates and it is therefore possible that the gene effects are not mediated (entirely) by total bilirubin. Such pleotropic effects at this loci are likely to be the case as evidenced by the fact that adjustment for serum bilirubin in our modelling did not completely nullify the observed association between genotype and outcome. Future work is required to more fully explore these effects along with associations of other substrates with variants at this genomic region.

Mutations in UGT1A1 have also been associated with Crigler-Najjar syndromes types I and II and in Gilbert syndrome [44–46]. Gilbert Syndrome (GS) is a well-documented benign increase in serum bilirubin, and is caused by the reduced activity of UDPGT [47–51]. In line with the observations that serum bilirubin is inversely correlated with metabolic risk diabetic patients with GS are less likely to develop vascular dysfunctions [52]. Furthermore, the incidence of diabetes and cardiovascular disease risk mortality is lower in GS individuals, with one study exploring the efficacy of increasing serum bilirubin in type-2 diabetic patients [53]. Further evidence confirming the protective role of circulating bilirubin for type-2 diabetes has been reported in a prospective study [19].

Significant difference has been identified between functional polymorphisms within the UGT1A family between Caucasian and other populations [54]. Polymorphisms in the promoter region for UGT1A1 (2 bp TA insertion in the TATA box) increased activity in Caucasian GS patients; this was not observed in Asian and African GS patients or Pacific populations [54]. The authors suggest that due to the complex nature of environmental and genetic factors, unstable polymorphisms within UGT1A1 may act to “fine-tune” plasma bilirubin levels on a population by population basis, meaning that the promoter variation explains the presence of GS in some populations, but in other populations it's more likely a combination of variants in the encoding region along with environmental factors [54], our data supports this hypothesis. Additionally, meta-analysis has demonstrated strong replication for a genetic influence on serum bilirubin levels of the UGT1A1 locus (*P* < 5 × 10^−324^), specifically at the proximal promoter region of UGT1A1 tagged by rs6742078 [40]. While we didn’t have genotype information for this SNP we were able to impute against the 1000 Genomes panel to extrapolate associations between the two studies. Using imputed information we were able to illustrate that there is tight LD between rs6742078 and the top associated SNP from our study, rs6744284 (r^2^ = 0.85), suggesting that the Norfolk Island cohort exhibit a similar genetic pattern of association.

We identified strong LD across the region of 2q37.1, potentially suggesting that the Norfolk Island population’s unique genomic structure is influencing serum bilirubin concentration. LD across the same region in data available through the HapMap project [55] showed that the Norfolk Island cohort exhibited an LD pattern similar to that observed in the European population (CEU), while both the Asian populations (Chinese and Japanese) exhibited very different genetic structure across this region. This is not unexpected because of the large amount of recent European admixture in the Norfolk population. Additionally, it was noted that haplotypes containing the minor allele(s) in the Norfolk Island population potentially conferred protection to metabolic disorders as measured by clinical metabolic syndrome and type-2 diabetes-risk. It is possible that selection is driving the presence of high serum bilirubin within populations, although this may be achieved by different variation across the region. It appears that in Europeans this variation is often in the promoter region, whereas in Asian and African populations this is not the case, and it is polymorphisms in the gene body that seems to account for the associations with increased bilirubin. This strongly suggests that it is beneficial for a population to have a certain frequency of individuals with naturally high serum bilirubin, and potentially points to a complex interaction between environmental and genomic factors maintaining this.

One significant association between 2 SNPs (rs2741012 and rs2741027) and categorised type-2 diabetes-risk was observed. These two SNPs are just upstream of the promoter and 5'UTR region of the UDPGT family. It is likely that these SNPs are in LD with untyped polymorphisms (SNPs not on the 610quad chip) that reside in these regions and potentially form a LD block/haplotype in the Norfolk Island population which confers protection to type-2 diabetes as well as metabolic syndrome. Interestingly, and in support of our approach, this reduction in risk correlates well with previous work conducted in a large US cohort [13]; these variants (or variants tagged by them) may be functional, i.e. they might directly affect transcription and/or translation of the isoforms encoded by the UDPGT family. It is also possible that there are additional rare variants within the region that further influence serum bilirubin as recently evidenced by an exome sequencing study performed in elderly individuals [56].

Given that bilirubin is a cheap and commonly measured laboratory test, routine screening of serum bilirubin levels could be beneficial in the stratification and treatment of metabolic disorders such as cardiovascular disease and type-2 diabetes. Identification of genes/variants that exhibit pleiotropic effects (effects of the same variant on multiple characteristics or disease risks) is an ultimate goal. The significant interaction observed here provides evidence that bilirubin may be affected by genetic and environmental factors and their interactions.

## Conclusions

In summary, this study identified strong associations of variants within the UGT1A family with regulation of serum bilirubin levels in the Norfolk Island population, which replicated previous GWAS and epidemiological findings. This successful implementation of pedigree-based analysis using the unique properties of the Norfolk Island cohort highlights a functional region that offers protective benefit from metabolic disease and further eludes to a potentially heritable component with the Norfolk Island population. Specific haplotype structure was significantly associated with increased serum bilirubin, and as such this study has identified a potential set of 'protective' haplotypes that exist within the Norfolk Island population. Further studies are warranted to validate these findings, with the next step being to explore these associations in larger outbred populations.

## Methods

### Sample/cohort collection, pedigree information and ethics

The Norfolk Island Health Study (NIHS) is well established with regards to data collection and initial disease prevalence studies [4, 5, 8]. The Norfolk Island pedigree structure has been previously outlined [57], and subsequently updated [1]. The most recent update led to the reconstruction of a core-pedigree consisting of 1388 members coalescing over 11 generations (or 200 years) back to the original founders. [3, 7]. This study focuses on a reduced core-pedigree, meaning that individuals; a) are genetically related to the original founders, and b) have phenotype and genotype information available. The total number of individuals fitting these criteria was 382. All individuals gave written informed consent. Ethical approval was granted prior to the commencement of the study by the Griffith University Human Research Ethics Committee (ethical approval no: 1300000485) and the project was carried out in accordance with the relevant guidelines, which complied with the Helsinki Declaration for human research.

### Heritability analysis

All 19 metabolic traits assessed in this analysis are part of the NIHS2000 collection [8]. Traits measured were: fasting plasma glucose, HDL-C, LDL-C, total plasma cholesterol, cholesterol-HDL-C ratio, triglycerides, creatinine, total protein, globin, albumin, urea, uric acid, total serum bilirubin, direct serum bilirubin, and numerous enzymes that are markers for liver/kidney function (ALT, AST, Alkaline Phosphatase, GGT, Lactate Dehydrogenase [LDH]).

### Heritability estimates

The R package and genetic analysis program GenABEL [58] was used to calculate heritability estimates for all metabolic syndrome/cardiovascular disease related traits. The genetic kinship matrix derived from the SNP data and reconstructed core-pedigree was used to estimate trait heritability (h^2^ [narrow-sense heritability]) by polygenic modelling. All traits were screened for covariant effects of age and sex interactions.

### Genome-wide SNP genotyping

EDTA anticoagulated venous blood samples were collected from all participants. Genomic DNA was extracted from blood buffy coats using standard phenol-chloroform procedures. DNA samples were genotyped according to the manufacturer’s instructions on Illumina Infinium High Density (HD) Human610-Quad DNA analysis BeadChip version 1. BeadChips were a four-sample format requiring 200 ng of DNA per sample. Samples were scanned on the Illumina BeadArray 500GX Reader. Raw data was obtained using Illumina BeadScan image data acquisition software (version 2.3.0.13). Raw data from Illumina idat files were SNP genotyped in R using the CRLMM package [59]. Genotype data then underwent initial quality control routines using PLINK [60]. SNPs were filtered based on: minor allele frequency >0.01; call rate >0.95, and Hardy-Weinberg equilibrium testing p-value >10^−5^. After this initial quality control, 590,603 SNPs were exported from PLINK and imported into the CRAN package GenABEL [58]. Further filtering (including Mendelian inheritance violations and sex-checking based on available X and Y markers) in GenABEL lead to the reduction of the SNP set to a total of ~480,000; this included removal of both X and Y chromosome SNPs after gender checking, as well as the removal of mitochondrial and XY SNPs.

### Genome-wide association analysis

A pedigree based GWAS analysis of all heritable traits was batched using custom R scripts and the package GenABEL [58]. GenABEL uses an additive approach and the loci are coded as 0, 1, 2 (corresponding to genotypes AA, AB, and BB, respectively). A detailed explanation of the association model and specific GWAS overview as implemented in the Norfolk Island was previously described [7]. Breifly, a correction was made for the relatedness inherent in the Norfolk Island population using the polygenic model with age and sex interactions, as well as genetic structure [the top 2 genomic principal components of the complete SNP set as calculated by KING [61]]. The top two components were chosen as covariates because we found that these explained the majority of the variance in the outcomes being tested and because inclusion of additional, less informative components only served to reduce the parsimony of the models. For association analysis the mmscore function implemented in GenABEL was used. This function represents a mixed model approximation analysis for association between a trait and genetic polymorphism(s), and is specifically designed for association testing in samples of related individuals. This allows for per SNP association testing using a mixed model polygenic approach. After correcting for multiple testing, the study-wide significance was set based on *M*_*eff*_ adjustment (P = 1.84 × 10^−7^). It should be noted that this *M*_*eff*_ threshold is tailored to trait-wise associations, not multi-trait analyses therefore p-values are adjusted on a per trait basis. Association statistics for every SNP for each trait were generated and output to compressed files (.gz.tar) for storage and future reference. GWAS Manhattan plots where generated for each trait association using a custom modified version of the GenABEL plot.scan.gwaa function (for all Manhattan plots see Additional file 2). Annotation of the robustly associated bilirubin SNPs identified as being functional was performed using: http://brainarray.mbni.med.umich.edu/Brainarray/Database/SearchSNP/snpfunc.aspx.

### LD testing and haplotype association

Genotype data for the chrq37.12 region was phased using SHAPEIT2 [62], which has functionality to deal with complex pedigree structures – implemented through the duoHMM algorithm. From this process we observed no Mendelian errors before moving the phased data over to Haploview analyses. Haplotype/LD testing, SNP tagging and association analyses were all conducted in Haploview 4.2 [63]. LD blocks were determined using the default Haploview settings which infer LD based on a pairwise comparison of correlation (r^2^) values between SNPs. Haplotypes were inferred from the genotypes of SNPs which made up the identified LD blocks, and were only recorded if they existed in more than 1 % of the population. Tagging SNPs were determined using the 'tagger' option of Haploview, using a pair-wise tagging method with a minimum observed r^2^ between pairs of 0.8. Association analyses were carried out on both markers (SNPs) and haplotypes using the inbuilt Haploview association function. A phenotype column was added to the dataset to allow a 'case'/'control' experimental set-up; where case represented the high bilirubin group and control the normal bilirubin group. There were a total of 65 cases and 317 controls with 124 genotyped individuals missing phenotype information. Permutation testing was run to confirm the above association analyses for both marker and haplotype associations. To ensure the robustness of final P values the number of permutations was set at 1,000,000 (this should lead to a reduction of the FDR). A further exploration of potentially similar structure across the region spanned by the 29 SNPs was tested in 3 HapMap populations; CEU (European), CHD (Chinese), and JPT (Japanese). Due to data being generated on different genotype platforms (SNP chips), a final list of 25 consensus SNPs was retained for Haploview analysis for Norfolk Island and the 3 HapMap sets. Linkage disequilibrium plots across the 25 SNP region was generated for each of the 4 populations (Additional file 3).

### Correlations with metabolic traits

Initial exploratory correlations between risk scores for cardiovascular disease and type-2 diabetes, clinically defined Metabolic Syndrome (categorical: 0 (no MetS), 1 (MetS)), and various related traits were conducted in R 2.15.2 [64]. For all analyses total serum bilirubin levels were categorised into 'normal' and 'high' groupings, with 'high' being defined as >14 μmol/L, this approximates a clinical cut-off and allows facilitates interpretation in line with existing clinical guidlines. For all other traits tested a standard student's *t*-test (as implemented in R) was used to test for a significant difference of means between the given trait and bilirubin level. There were two categorical traits tested for correlation with serum bilirubin levels; smoking and presence of metabolic syndrome. Smoking has been previously well documented to be associated with serum bilirubin levels [23], and was categorised in the Norfolk Island cohort as either 'yes' (smokers *N* = 133) or 'no' (non-smokers *N* = 458). Correlation testing between smoking and bilirubin was carried out using a 2 × 2 chi-squared contingency test, followed by a Fisher's Exact test. For correlation analysis between total serum bilirubin and metabolic syndrome there were a total of 598 individuals with available matched phenotype data; 'metabolic syndrome' (*N* = 156) and 'no metabolic syndrome' (*N* = 442). The clinical diagnosis of metabolic syndrome previously calculated for the Norfolk Island cohort was used [7]. A 2 × 2 chi-squared contingency test was used to evaluate the significance, followed by a Fisher's Exact test as one of the tables cells contained a value less than 5 %. Due to the initial exploratory nature of these analyses all tests are unadjusted, so nominal p-values are reported. Additionally, relatedness within the population is accounted for in later formal modelling using GLM regression.

### Regression modelling testing association between outcome, trait and genotype

To further explore associations between bilirubin, type-2 diabetes and the genotypic architecture across the *UGT1A1* region regression modelling was conducted in R. To establish an initial association, separate logistic regression was conducted between categorised type-2 diabetes risk and total bilirubin and then direct bilirubin. Additionally a bi-directional stepwise logistic regression model was used to test the significance of each of the 9 tagging SNPs identified in the LD block analysis. The model was not corrected for common covariates (age, sex, smoking, BMI) as these are all accounted for in the calculation of the AUSDRISK type-2 diabetes risk score (as previously calculated in the Norfolk Island cohort [7]). To address the issue of relatedness we included the average pedigree kinship as a covariate in the stepwise regression model. This was excluded from the final model indicating that in this instance relatedness is not a significant issue. Reported r^2^ values use the Nagelkerke Index pseudo r^2^ as calculated in R. Model p values were generated from an ANOVA using the F distribution, which tests the null hypothesis that the coefficients represented in the overall regression model (represented by R 2) are equal to 0.

### Web resources

Online Mendelian Inheritance in Man (http://www.omim.org)

Catalogue of genome-wide association studies (http://www.genome.gov/gwastudies)

BrainArray (http://brainarray.mbni.med.umich.edu/Brainarray/Database/SearchSNP/snpfunc.aspx).

### Availability of supporting data

Due to current ethical constraints, restricted data access is in place to anonymise genotypic SNP GWAS and phenotype data. The Norfolk Island Health Study steering committee will assess restricted data access requests via our GRC computational genetics group (interested researchers should contact grccomputationalgenomics@gmail.com).

## Additional files

Additional file 1:
**Summary statistics of phenotypic traits measured in the Norfolk Island population.** Table with an overview of the Norfolk Island phenotype data analysed, 16 traits in total. (PDF 96 kb) Additional file 2:
**GWAS Manhattan plots for metabolic related traits.** GWAS Manhattan plots for all 16 traits. (PDF 7896 kb) Additional file 3:
**LD lots for 4 populations across 200 kb of chr2q37.1.** Haploview LD plots for 25 SNPs spanning a region of chr2q37.1 for four populations; NI (Norfolk Island), CEU (European); CHD (Chinese), and JPT (Japanese). (PDF 2809 kb) Additional file 4:
**Haploview allele frequency data for 25 chr2q37.1 SNPs across 4 populations.** Minor allele frequencies for 25 SNPs across five populations; Norfolk Island (NI), European (CEU), Chinese (CHD and CHB), and Japanese (JPT). (PDF 137 kb) Additional file 5:
**Detailed Haploview allele frequency data for all 29 SNPs in the NI cohort.** Allele frequency data for all 29 SNPs across the chr2q37.1 region for the Norfolk Island samples. (PDF 155 kb) Additional file 6:
**Haploview haplotype associations with bilirubin levels.** Haplotypes identified by Haploview and their association statistics. (PDF 33 kb)

## Abbreviations

BMI: Body mass index
CEU: European Hapmap population
CHD: Chinese Hapmap population
CVD: Cardiovascular disease
DBP: Diastolic blood pressure
GS: Gilbert’s syndrome
GWAS: Genome-wide association study
h^2^: Heritability (narrow-sense)
HDL-C: High-density lipoprotein cholesterol
JPT: Japanese Hapmap population
LD: Linkage disequilibrium
LDL-C: Low-density lipoprotein cholesterol
MAF: Minor allele frequency
NI: Norfolk Island
NIHS: Norfolk Island health study
SBP: Systolic blood pressure
SNP: Single nucleotide polymorphism
