# Supplementary material for: Serum bilirubin concentration is modified by UGT1A1 Haplotypes and influences risk of Type-2 diabetes in the Norfolk Island genetic isolate
Source: BMC Genet. 2015 Dec 2;16:136. doi: 10.1186/s12863-015-0291-z (PMC4667444; doi:10.1186/s12863-015-0291-z)
Supplement: Additional file 1: — Summary statistics of phenotypic traits measured in the Norfolk Island population. Table with an overview of the Norfolk Island phenotype data analysed, 16 traits in total. (PDF 96 kb) [file 12863_2015_291_MOESM1_ESM.pdf]

| Trait | Total (mean) | Std dev | Male (mean) | Std dev | Female (mean) | Std dev |
|---|---|---|---|---|---|---|
| Glucose | 5.62 | 0.96 | 5.77 | 0.99 | 5.48 | 0.92 |
| Cholesterol | 5.64 | 1.11 | 5.81 | 1.09 | 5.50 | 1.11 |
| Triglycerides | 1.99 | 1.17 | 2.29 | 1.23 | 1.72 | 1.05 |
| Chol/HDL ratio | 4.32 | 1.33 | 4.83 | 1.31 | 3.89 | 1.19 |
| HDL-C | 1.38 | 0.34 | 1.26 | 0.29 | 1.49 | 0.35 |
| LDL-C | 2.85 | 0.97 | 3.03 | 0.94 | 2.70 | 0.97 |
| Albumin | 41.54 | 2.73 | 42.11 | 2.76 | 41.05 | 2.62 |
| Globin | 29.64 | 3.45 | 29.65 | 3.66 | 29.63 | 3.28 |
| Total protein | 71.18 | 4.66 | 71.75 | 5.02 | 70.69 | 4.27 |
| LDH | 124.46 | 34.53 | 126.04 | 31.58 | 123.11 | 36.91 |
| Creatinine | 81.29 | 16.75 | 89.40 | 16.22 | 74.38 | 13.88 |
| Total Bilirubin | 7.59 | 4.19 | 8.25 | 4.20 | 7.02 | 4.10 |
| Direct Bilirubin | 2.55 | 1.43 | 2.68 | 1.47 | 2.45 | 1.40 |
| Alk Phos | 63.47 | 18.34 | 68.07 | 19.21 | 59.53 | 16.62 |
| GGT | 27.97 | 29.23 | 36.92 | 34.34 | 20.29 | 21.26 |
| ALT | 18.13 | 11.61 | 21.46 | 12.15 | 15.27 | 10.34 |
| AST | 21.37 | 9.36 | 23.55 | 9.54 | 19.50 | 8.81 |
| Uric acid | 0.34 | 0.09 | 0.40 | 0.08 | 0.30 | 0.08 |
| Urea | 5.65 | 1.50 | 6.07 | 1.32 | 5.28 | 1.56 |
