# Supplementary figures and images for: Serum bilirubin concentration is modified by UGT1A1 Haplotypes and influences risk of Type-2 diabetes in the Norfolk Island genetic isolate
Source: BMC Genet. 2015 Dec 2;16:136. doi: 10.1186/s12863-015-0291-z (PMC4667444; doi:10.1186/s12863-015-0291-z)

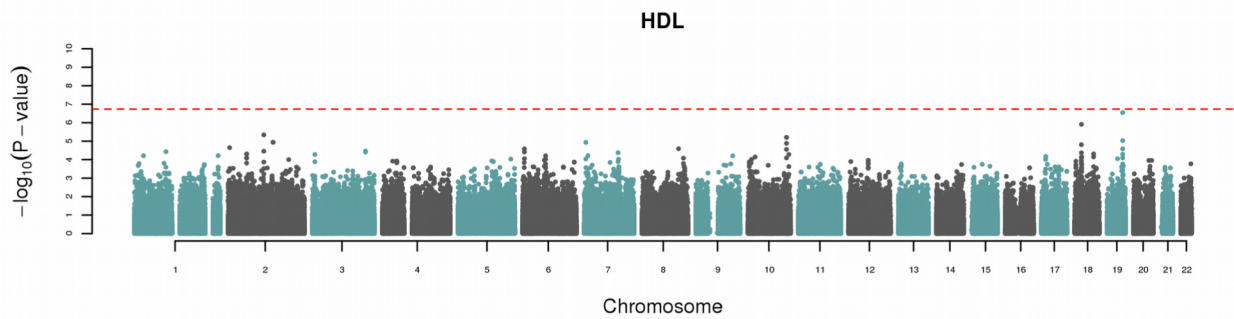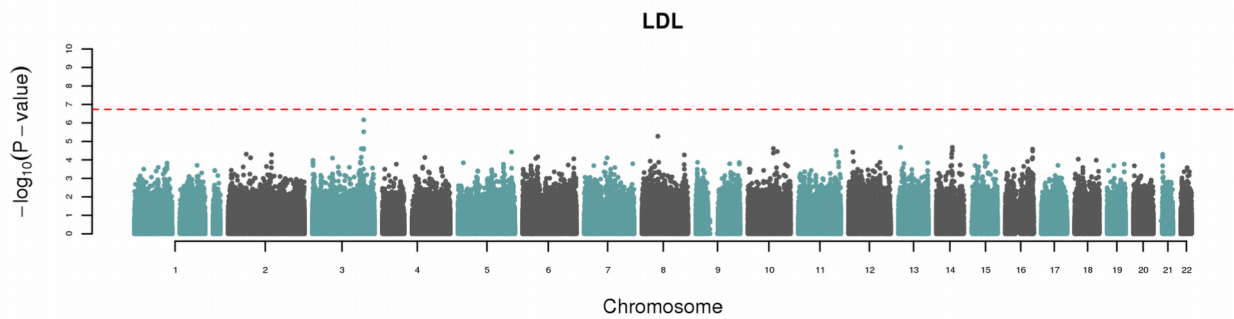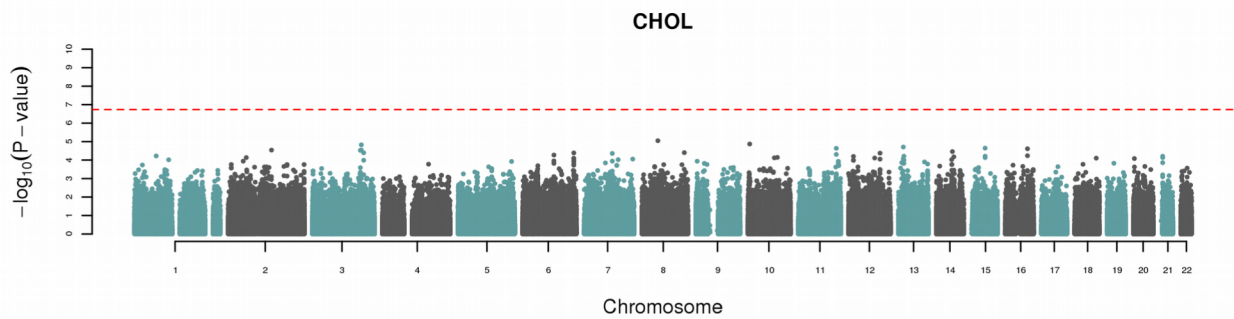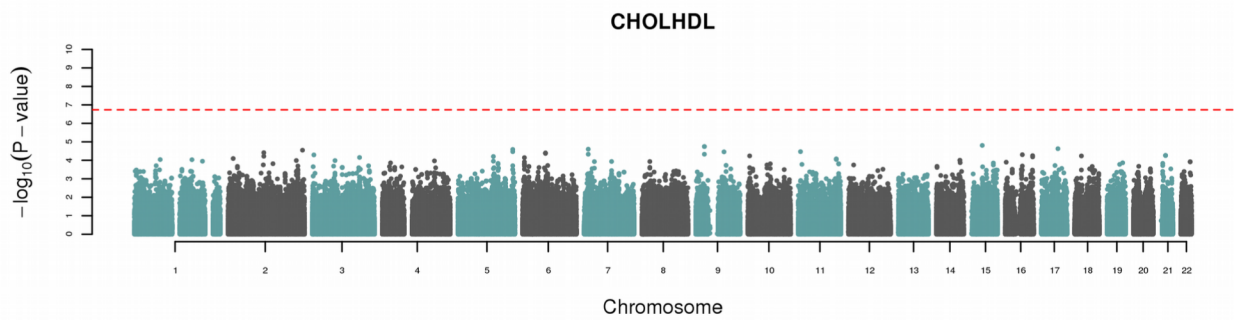

### T\_BILI

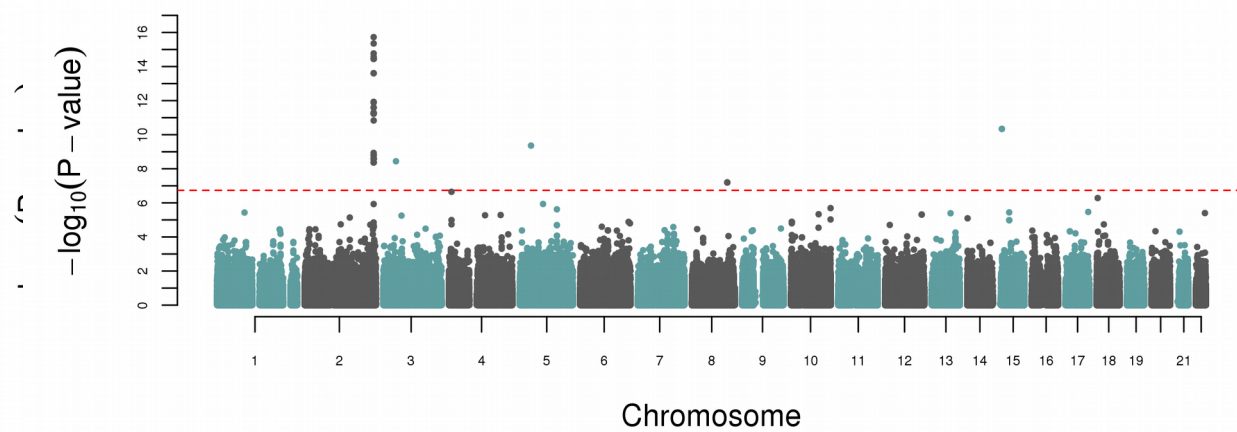

### D\_BILI

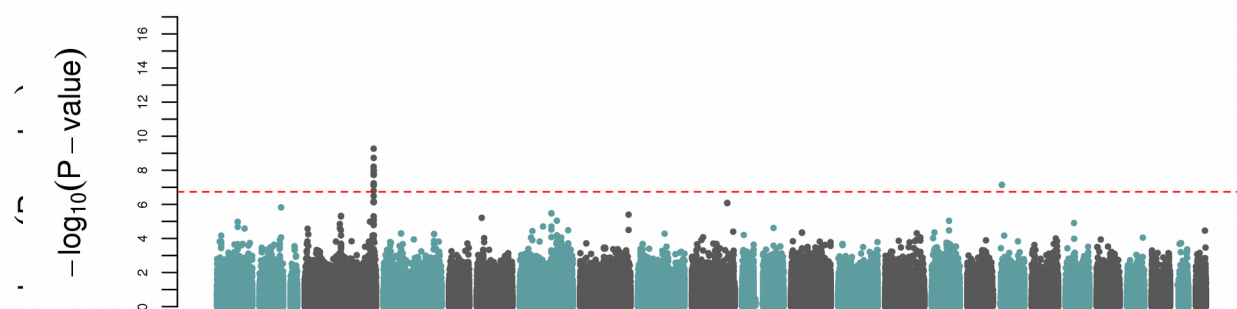

### CREAT

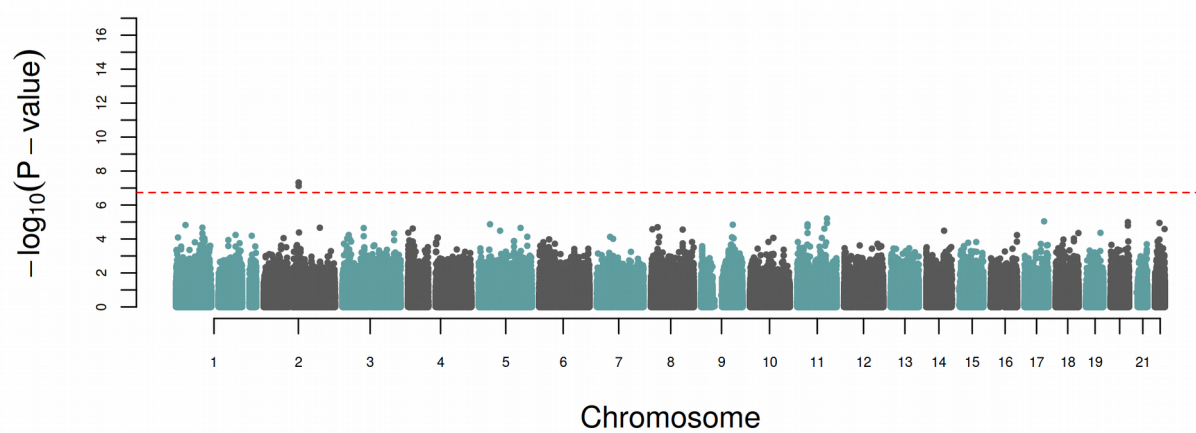

### CHLOR

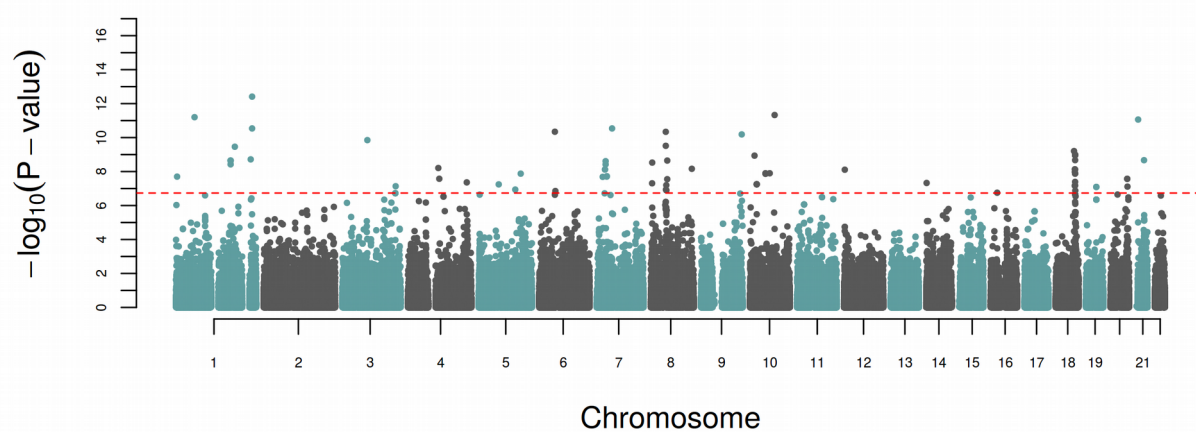

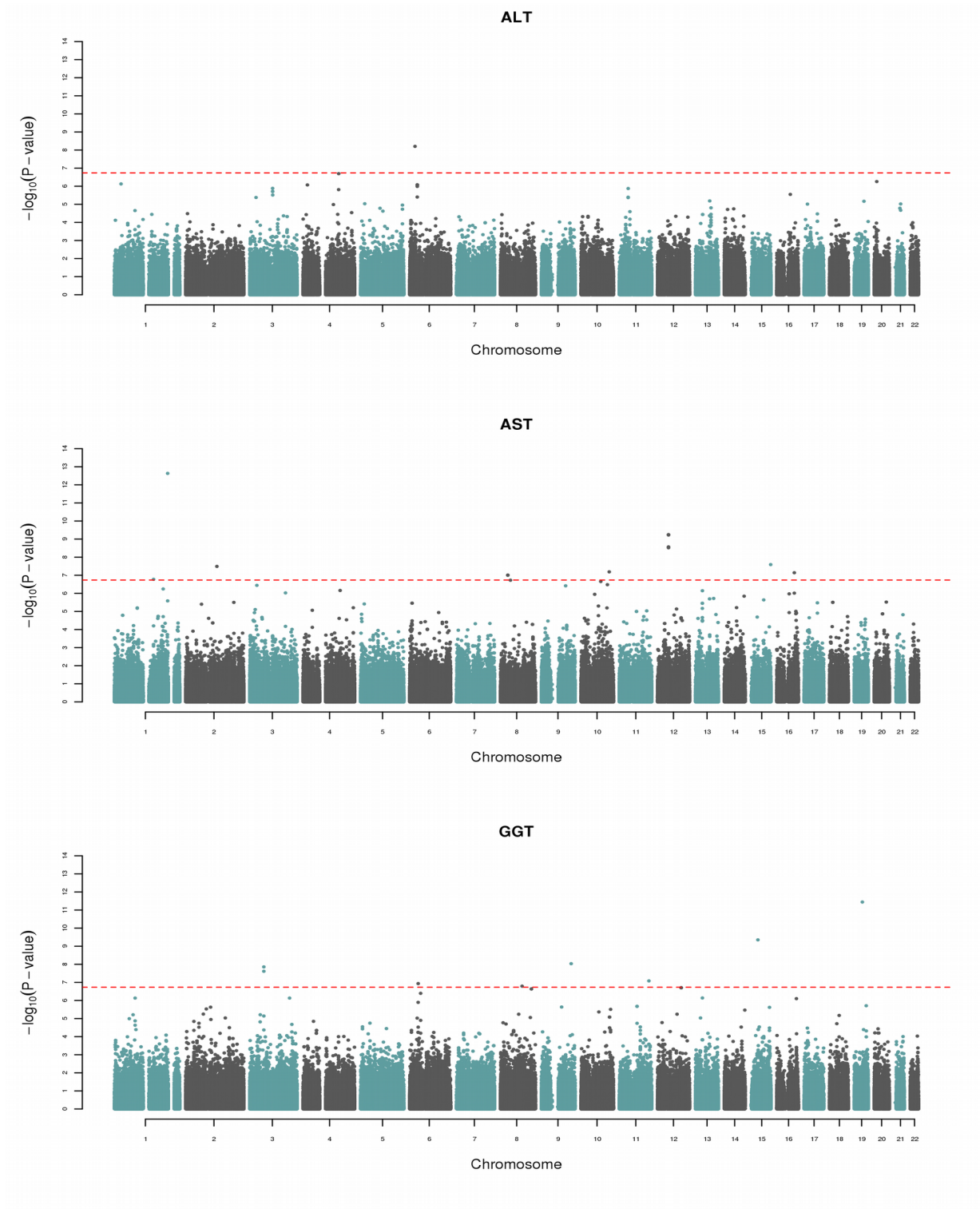

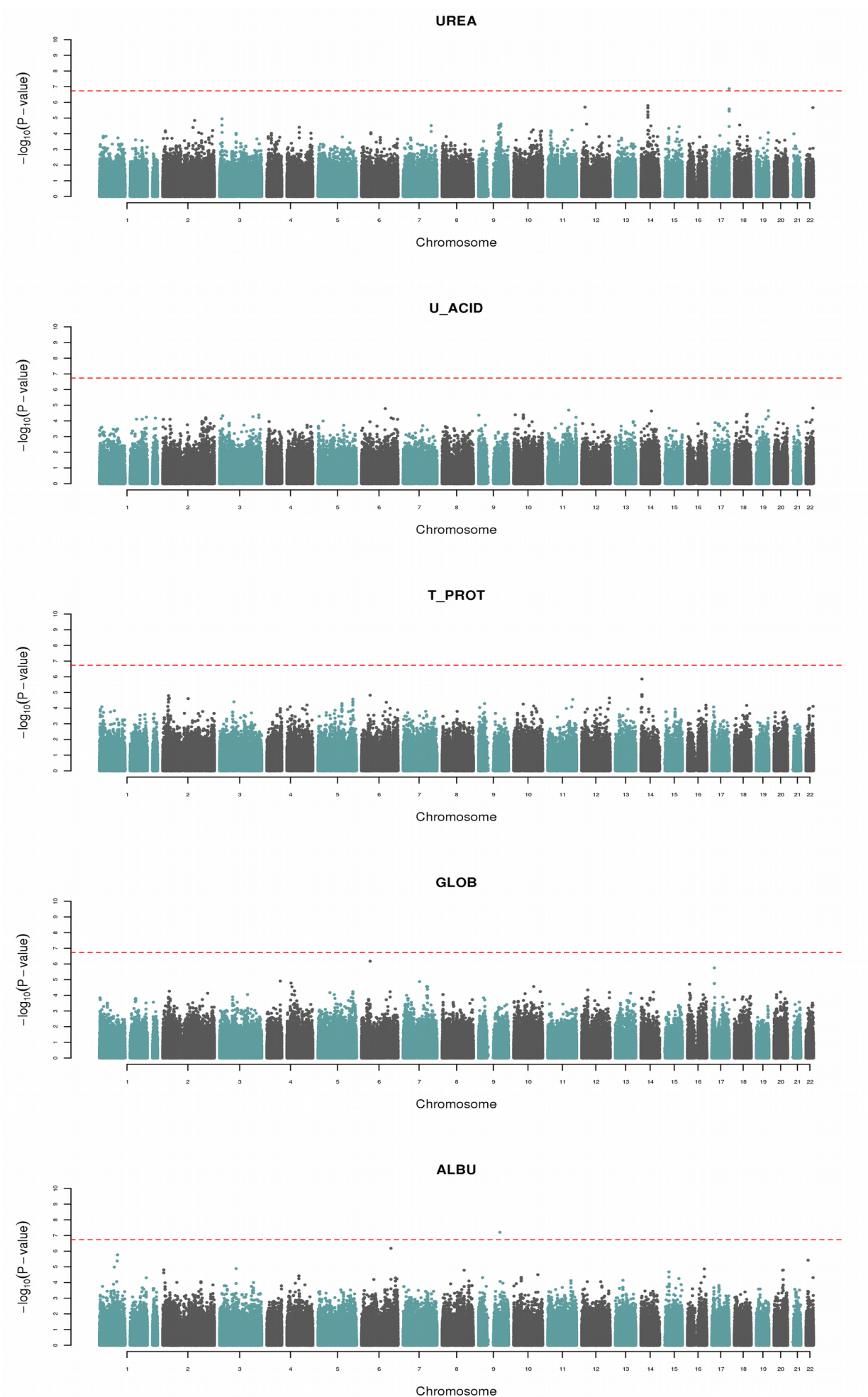

Supplement: Additional file 2: — GWAS Manhattan plots for metabolic related traits. GWAS Manhattan plots for all 16 traits. (PDF 7896 kb) [file 12863_2015_291_MOESM2_ESM.pdf]

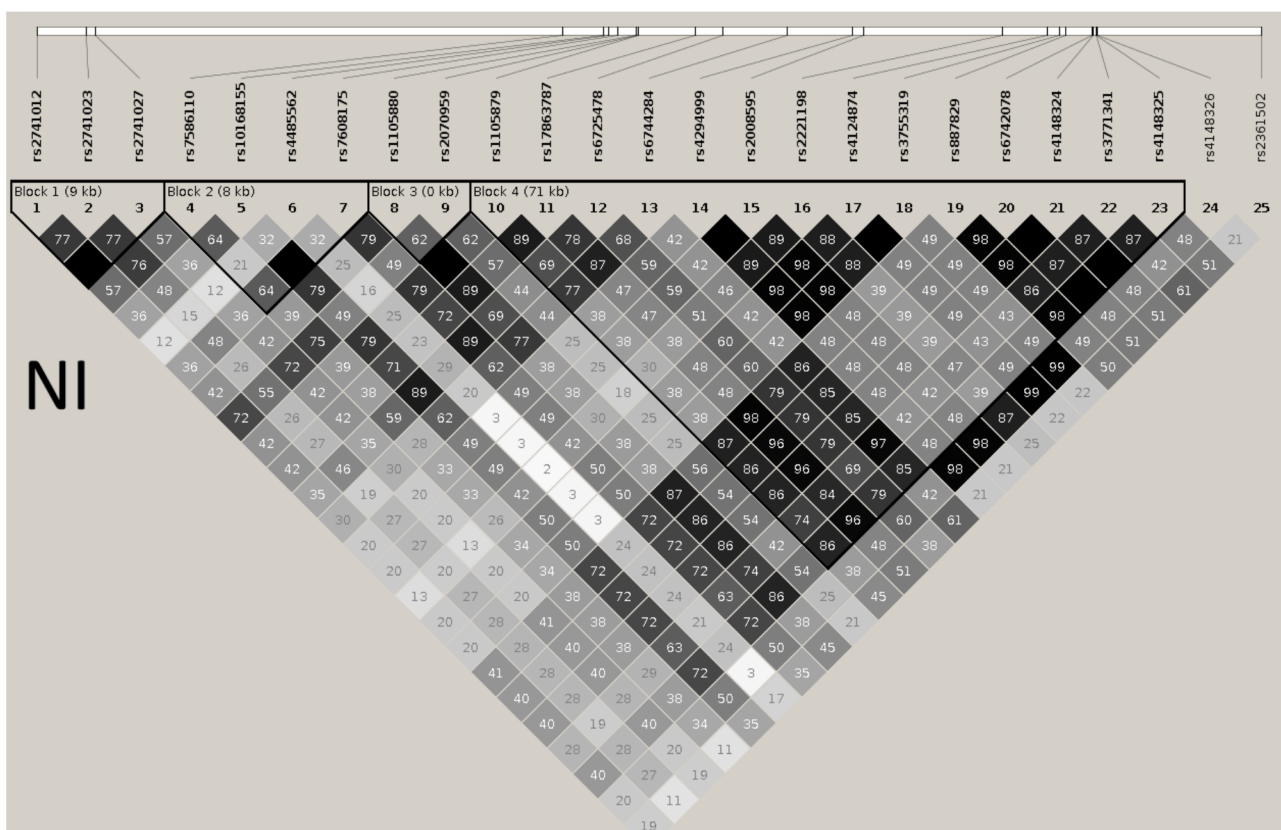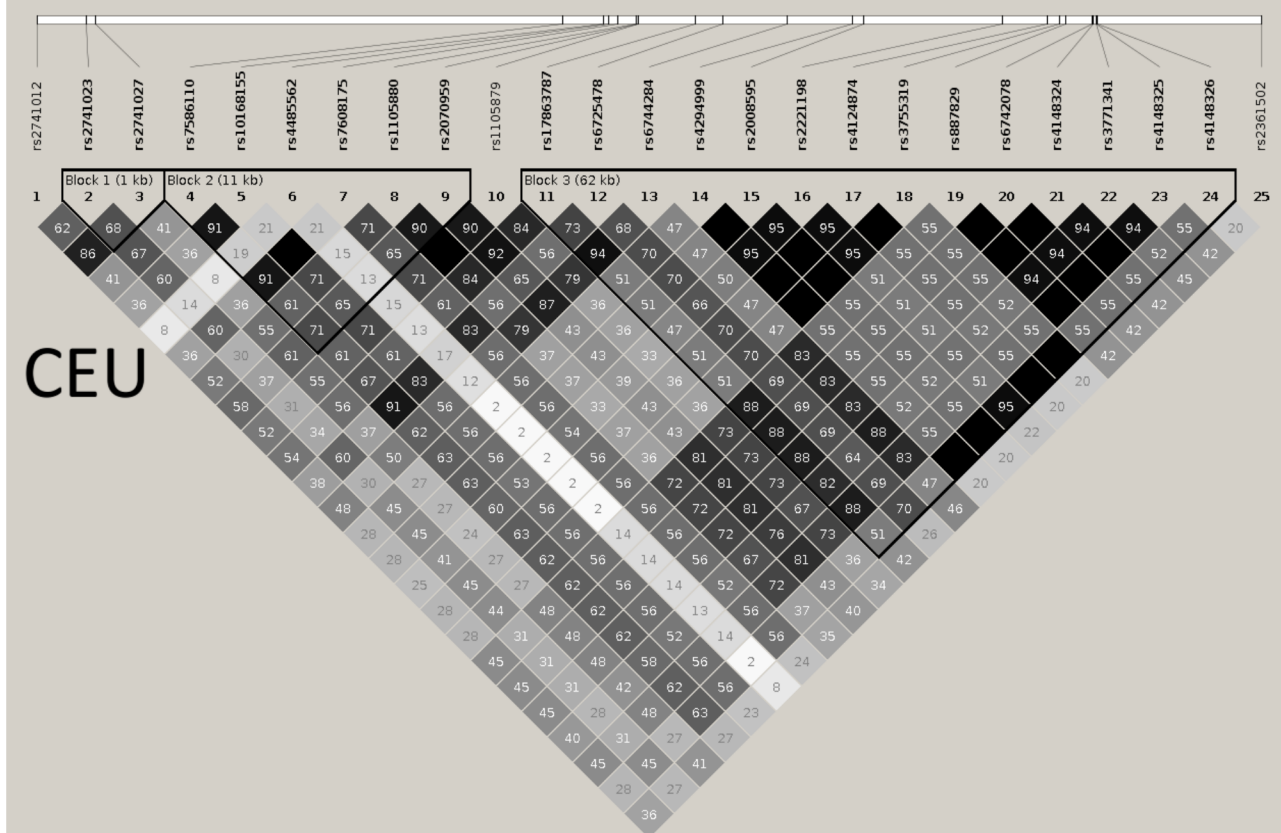

CHD

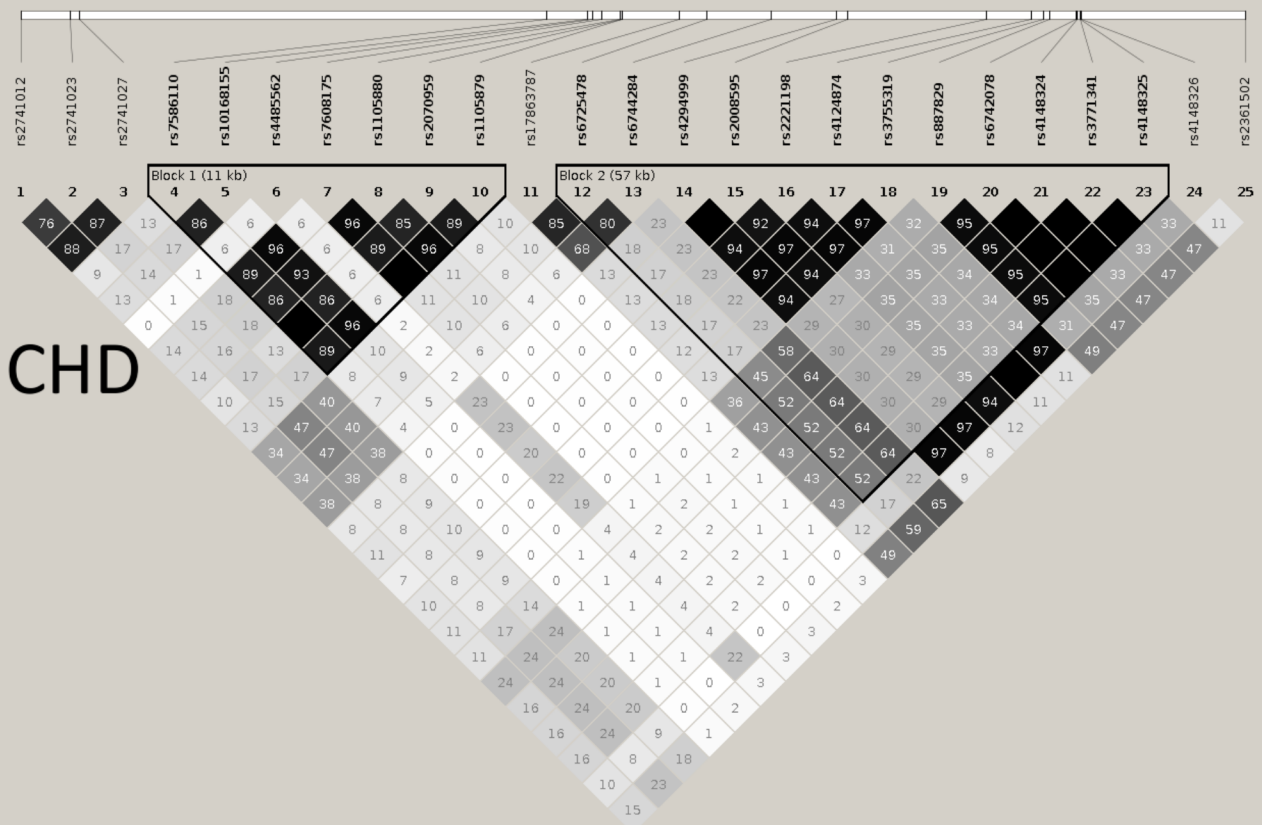

JPT

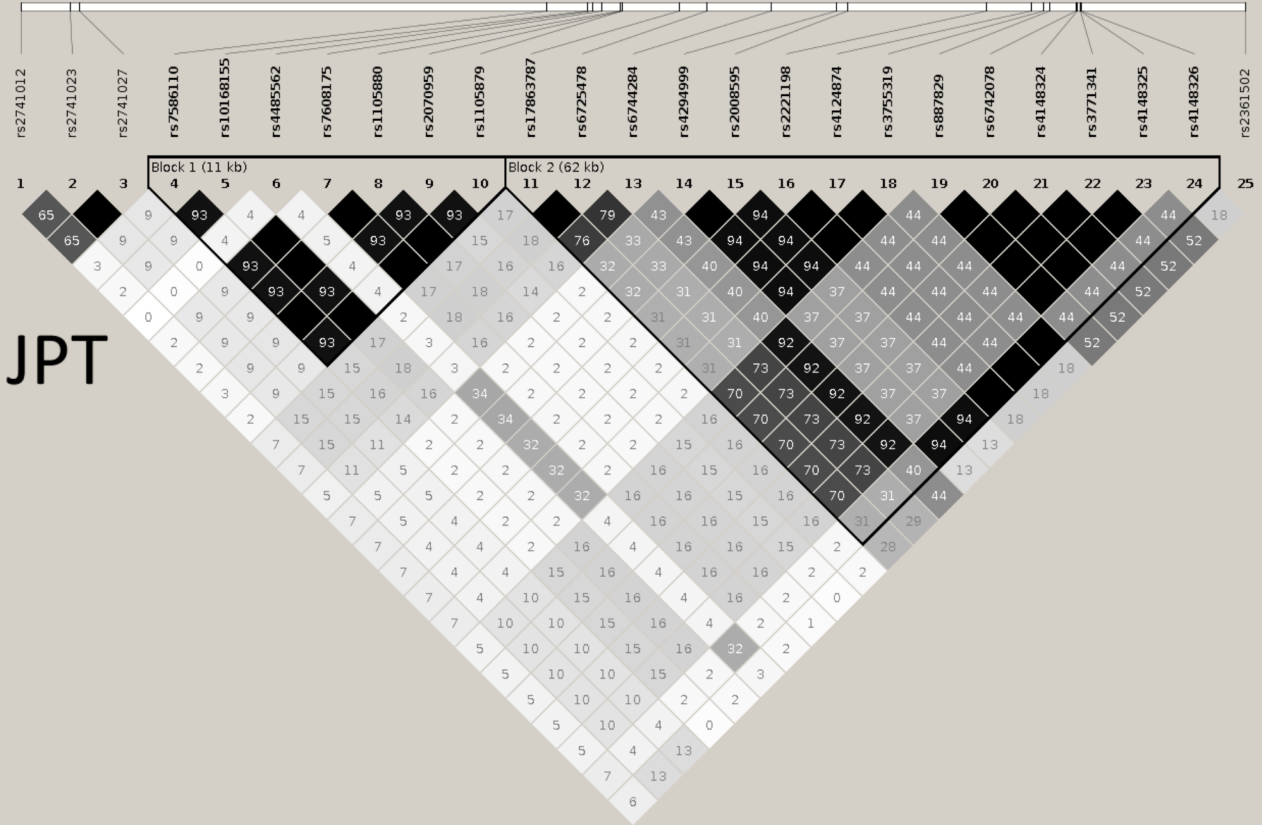

Supplement: Additional file 3: — LD lots for 4 populations across 200 kb of chr2q37.1. Haploview LD plots for 25 SNPs spanning a region of chr2q37.1 for four populations; NI (Norfolk Island), CEU (European); CHD (Chinese), and JPT (Japanese). (PDF 2809 kb) [file 12863_2015_291_MOESM3_ESM.pdf]
