# Supplementary material for: Serum bilirubin concentration is modified by UGT1A1 Haplotypes and influences risk of Type-2 diabetes in the Norfolk Island genetic isolate
Source: BMC Genet. 2015 Dec 2;16:136. doi: 10.1186/s12863-015-0291-z (PMC4667444; doi:10.1186/s12863-015-0291-z)
Supplement: Additional file 4: — Haploview allele frequency data for 25 chr2q37.1 SNPs across 4 populations. Minor allele frequencies for 25 SNPs across five populations; Norfolk Island (NI), European (CEU), Chinese (CHD and CHB), and Japanese (JPT). (PDF 137 kb) [file 12863_2015_291_MOESM4_ESM.pdf]

Name	Position	Alleles	MAF_NI	MAF_CEU	MAF_CHD	MAF_CHB	MAF_JPT
rs2741012	234508963	C:T	0.23	0.29	0.05	0.04	0.04
rs2741023	234516714	G:A	0.27	0.34	0.04	0.03	0.02
rs2741027	234518011	G:A	0.23	0.26	0.05	0.03	0.02
rs7586110	234590527	T:G	0.29	0.38	0.20	0.26	0.20
rs10168155	234596836	C:T	0.38	0.40	0.22	0.27	0.21
rs4485562	234597566	G:A	0.35	0.25	0.19	0.17	0.16
rs7608175	234599089	C:G	0.38	0.40	0.21	0.27	0.21
rs1105880	234601965	A:G	0.33	0.32	0.21	0.27	0.21
rs2070959	234602191	A:G	0.23	0.30	0.20	0.26	0.20
rs1105879	234602202	A:C	0.33	0.32	0.22	0.27	0.21
rs17863787	234611094	T:G	0.31	0.29	0.08	0.04	0.13
rs6725478	234615400	C:T	0.36	0.36	0.08	0.04	0.14
rs6744284	234625297	C:T	0.29	0.27	0.10	0.09	0.17
rs4294999	234635467	A:G	0.49	0.44	0.33	0.29	0.32
rs2008595	234637192	C:T	0.49	0.44	0.34	0.29	0.32
rs2221198	234658623	G:A	0.46	0.43	0.33	0.30	0.33
rs4124874	234665659	T:G	0.48	0.44	0.34	0.30	0.33
rs3755319	234667582	A:C	0.48	0.44	0.34	0.30	0.33
rs887829	234668570	C:T	0.32	0.30	0.14	0.11	0.18
rs6742078	234672639	G:T	0.31	0.30	0.15	0.11	0.18
rs4148324	234672722	T:G	0.31	0.30	0.15	0.11	0.18
rs3771341	234673239	C:T	0.29	0.29	0.15	0.11	0.18
rs4148325	234673309	C:T	0.31	0.30	0.15	0.11	0.18
rs4148326	234673462	T:C	0.48	0.44	0.34	0.30	0.33
rs2361502	234698790	T:C	0.31	0.26	0.11	0.14	0.15

Note: MAF (minor allele frequency)
