# Supplementary material for: Serum bilirubin concentration is modified by UGT1A1 Haplotypes and influences risk of Type-2 diabetes in the Norfolk Island genetic isolate
Source: BMC Genet. 2015 Dec 2;16:136. doi: 10.1186/s12863-015-0291-z (PMC4667444; doi:10.1186/s12863-015-0291-z)
Supplement: Additional file 5: — Detailed Haploview allele frequency data for all 29 SNPs in the NI cohort. Allele frequency data for all 29 SNPs across the chr2q37.1 region for the Norfolk Island samples. (PDF 155 kb) [file 12863_2015_291_MOESM5_ESM.pdf]

Name	Position (bp)*	ObsHET	PredHET	HWpval	MAF	Alleles
rs2741012	234508963	0.35	0.35	1.00	0.23	C:T
rs2741023	234516714	0.40	0.40	1.00	0.27	G:A
rs2741027	234518011	0.35	0.35	1.00	0.23	G:A
rs7586110	234590527	0.40	0.41	0.59	0.29	T:G
rs10168155	234596836	0.49	0.47	0.51	0.38	C:T
rs10168416	234597087	0.34	0.36	0.34	0.23	C:G
rs4485562	234597566	0.44	0.45	0.50	0.35	G:A
rs10171367	234597667	0.49	0.47	0.48	0.38	C:G
rs10179094	234597825	0.40	0.41	0.55	0.29	T:A
rs7608175	234599089	0.49	0.47	0.51	0.38	C:G
rs1105880	234601965	0.44	0.44	0.92	0.33	T:C
rs2070959	234602191	0.34	0.36	0.34	0.23	A:G
rs1105879	234602202	0.44	0.44	0.92	0.33	T:G
rs17863787	234611094	0.43	0.43	0.94	0.31	T:G
rs6725478	234615400	0.49	0.46	0.28	0.36	C:T
rs6744284	234625297	0.42	0.41	0.77	0.29	C:T
rs4294999	234635467	0.54	0.50	0.08	0.49	A:G
rs2008595	234637192	0.54	0.50	0.08	0.49	G:A
rs4663963	234650193	0.54	0.50	0.08	0.49	T:G
rs2221198	234658623	0.55	0.50	0.02	0.46	C:T
rs4124874	234665659	0.55	0.50	0.04	0.48	A:C
rs3755319	234667582	0.55	0.50	0.04	0.48	T:G
rs887829	234668570	0.44	0.43	0.73	0.32	G:A
rs6742078	234672639	0.44	0.43	0.82	0.31	G:T
rs4148324	234672722	0.44	0.43	0.78	0.31	T:G
rs3771341	234673239	0.42	0.41	0.68	0.29	C:T
rs4148325	234673309	0.44	0.43	0.78	0.31	C:T
rs4148326	234673462	0.55	0.50	0.05	0.48	T:C
rs2361502	234698790	0.42	0.43	0.70	0.31	T:C

*physical position is based upon build 37 (GRCh37) of the human genome.
