# Supplementary material for: Serum bilirubin concentration is modified by UGT1A1 Haplotypes and influences risk of Type-2 diabetes in the Norfolk Island genetic isolate
Source: BMC Genet. 2015 Dec 2;16:136. doi: 10.1186/s12863-015-0291-z (PMC4667444; doi:10.1186/s12863-015-0291-z)
Supplement: Additional file 6: — Haploview haplotype associations with bilirubin levels. Haplotypes identified by Haploview and their association statistics. (PDF 33 kb) [file 12863_2015_291_MOESM6_ESM.pdf]

**Block 1 – Haplotype**	**Freq.**	**High Bili Allele Freq**	**Normal Bili Allele Freq**	**OR (95% CI)**	**Chi Square**	**P Value**
CGGTCCACT	0.336	0.190	0.369	2.54 (1.58 – 4.07)	15.27	9.31E-05
CGGTCCGCT	0.262	0.135	0.289	2.65 (1.54 – 4.53)	13.12	3.00E-04
TAAGTGGGA	0.203	0.403	0.172	0.31 (0.20 – 0.46)	34.36	4.59E-09
CGGTTCGGT	0.096	0.155	0.078	0.46 (0.26 – 0.80)	7.81	5.20E-03
CAGGTCGGA	0.044	0.008	0.051	6.83 (0.92 – 50.4)	4.75	2.93E-02
CGGGTGGGA	0.030	0.094	0.021	0.20 (0.09 – 0.46)	17.13	3.49E-05
**Block 2 – Haplotype**						
CAAATCCACTGTACGTCCT	0.492	0.261	0.546	3.39 (2.23 – 5.17)	34.88	3.51E-09
GGGCGTTGTGAGCTTGTTC	0.188	0.435	0.143	0.22 (0.14 – 0.33)	58.82	1.73E-14
CAAATCCGTGAGCCGTCCC	0.120	0.085	0.127	1.57 (0.82 – 3.04)	1.81	1.79E-01
GGACGTTGTGAGCTTGTTC	0.089	0.142	0.072	0.47 (0.26 – 0.85)	6.93	8.50E-03
GAAATTCGTGAGCCGTCCC	0.048	0.008	0.047	6.43 (0.87 – 47.6)	4.37	3.66E-02
GGGCGTCGTGGGCTTGCTC	0.028	0.046	0.027	0.57 (0.22 – 1.48)	1.37	2.43E-01
GGGCTCCACTGTACGTCCT	0.015	0.012	0.021	2.30 (0.35 – 20.8)	0.54	4.62E-01

Note: odds ratios are not adjusted for age and sex
